# Therapeutic Management of *Pseudomonas aeruginosa* Bloodstream Infection Non-Susceptible to Carbapenems but Susceptible to “Old” Cephalosporins and/or to Penicillins

**DOI:** 10.3390/microorganisms6010009

**Published:** 2018-01-16

**Authors:** Ronit Zaidenstein, Asaf Miller, Ruthy Tal-Jasper, Hadas Ofer-Friedman, Menachem Sklarz, David E. Katz, Tsillia Lazarovitch, Paul R. Lephart, Bethlehem Mengesha, Oran Tzuman, Mor Dadon, Chen Daniel, Jacob Moran-Gilad, Dror Marchaim

**Affiliations:** 1 Unit of Infectious Diseases, Assaf Harofeh Medical Center, Zerifin 70300, Israel; ronitz@asaf.health.gov.il (R.Z.); ruthyanat@gmail.com (R.T.-J.); hadasofe@gmail.com (H.O.-F.); bettytade@gmail.com (B.M.); orantzuman@gmail.com (O.T.); mord@asaf.health.gov.il (M.D.); chendaniel77@gmail.com (C.D.); 2 Sackler School of Medicine, Tel-Aviv University, Tel-Aviv 6997801, Israel; 3 Intensive-Care Unit, Assaf Harofeh Medical Center, Zerifin 70300, Israel; asafmiller@gmail.com; 4 Bioinformatics Unit, National Institute for Biotechnology in the Negev, Beer-Sheva 84105, Israel; Sklarz@bgu.ac.il; 5 Department of Internal Medicine, Shaare Zedek Medical Center, Hebrew-University, Hadassah Medical School, Jerusalem 91031, Israel; dekatz1@gmail.com; 6 Clinical Microbiology Laboratory, Assaf Harofeh Medical Center, Zerifin 70300, Israel; zlazarovitch@asaf.health.gov.il; 7 Department of University Laboratories, Detroit Medical Center, Detroit, MI 48201, USA; drlephart@gmail.com; 8 Department of Health Systems Management, Faculty of Health Sciences, Ben-Gurion University of the Negev, Beer-Sheva 84105, Israel; giladko@post.bgu.ac.il; 9 Public Health Services, Ministry of Health, Jerusalem 91010, Israel; 10 ESCMID Study Group for Genomic and Molecular Diagnostics (ESGMD), 214 4010 Basel, Switzerland

**Keywords:** gram-negative, MDRO, non-fermenter, BSI, nosocomial infections, ICU

## Abstract

It is unknown as to whether other beta-lactams can be used for bloodstream infections (BSI) resulting from *Pseudomonas aeruginosa* (PA) which are non-susceptible to one or more carbapenem. We conducted a retrospective cohort study at the Assaf Harofeh Medical Center (AHMC) from January 2010 to August 2014. Adult patients with PA-BSI non-susceptible to a group 2 carbapenem but susceptible to ceftazidime or piperacillin (with or without tazobactam), were enrolled. We compared the outcomes of patients who received an appropriate beta-lactam antibiotic (“cases”) to those who received an appropriate non-beta-lactam antibiotic (“controls”). Whole genome sequencing was performed for one of the isolates. Twenty-six patients with PA-BSI met inclusion criteria: 18 received a beta-lactam and 8 a non-beta-lactam (three a fluoroquinolone, two colistin, one a fluoroquinolone and an aminoglycoside, one a fluoroquinolone and colistin, and one colistin and an aminoglycoside). All clinical outcomes were similar between the groups. There were large variations in the phenotypic susceptibilities of the strains. A detailed molecular investigation of one isolate revealed a strain that belonged to MLST-137, with the presence of multiple efflux pumps, OXA-50, and a chromosomally mediated Pseudomonas-derived cephalosporinase (PDC). The oprD gene was intact. Non-carbapenem-β-lactams may still be effective alternatives for short duration therapy (up to 14 days) for BSI caused by a carbapenem non-susceptible (but susceptible to ceftazidime, piperacillin, and/or piperacillin-tazobactam) PA strain. This observation requires further confirmatory analyses. Future molecular investigations should be performed, in order to further analyze additional potential mechanisms for this prevalent phenotype.

## 1. Introduction

*Pseudomonas aeruginosa* (PA) is a leading nosocomial pathogen causing various infectious syndromes [1,2,3]. PA is also notorious for its extensive intrinsic, adaptive and acquired resistance profiles to various antimicrobial agent classes [4]. Timely appropriate antibiotic treatment is therefore vital as PA infections are associated with devastating outcomes and high mortality rates [5].

Anti-Pseudomonal beta-lactam agents (e.g., penicillins, cephalosporins, carbapenems) are the agents of choice for PA infections, particularly for severe invasive PA infections, as long as the isolates are susceptible [3]. They are among the oldest, safest, cheapest (usually) agents available, and are highly bactericidal [3,6,7]. The major mechanisms of resistance to beta-lactams among PA isolates include beta-lactamase production, mutations that lead to low permeability of the bacteria outer membrane, and efflux pumps [4]. Some of the mechanisms are effective against the whole spectrum of beta-lactam agents, while others, are effective only against a few of the beta-lactams [4]. What further complicates the issue is the rapid selection and expression of certain resistance mechanisms while patients are treated with antimicrobials [4].

It is not known if infections caused by PA isolates resistant to group 2 carbapenems, can be treated with “older” beta-lactams such as ceftazidime and piperacillin (with or without tazobactam). It may be beneficial to use a bactericidal beta-lactam; however, emergence of resistance to those beta-lactams while the patient is on therapy is a real concern (i.e., particularly since the strains are already resistant to a carbapenem). Another option is to use a non-beta-lactam alternative (e.g., fluoroquinolones, aminoglycosides, colistin). These agents are usually less clinically effective, and some are potentially toxic with narrow therapeutic windows [3]. This clinical dilemma is especially relevant to countries where newer anti-Pseudomonal beta-lactams (e.g., ceftolozane-tazobactam and ceftazidime-avibactam) are not yet widely available or are too expensive. 

This PA phenotypic resistance pattern is becoming more prevalent. In Assaf Harofeh Medical Center, Israel, during 2013, these isolates represented 10.1% of all patient-unique PA bloodstream infections (BSI) strains. In the Detroit Medical Center, a 2200-bed healthcare system in Michigan, USA, during 2015, 13.5% (10 of 74) of its PA strains causing BSI, demonstrated these phenotypic features. Our goal was to conduct an efficacy analysis comparing non-carbapenem beta-lactams to non-beta-lactams, for the treatment of carbapenem-non-susceptible PA BSI, and to analyze the mechanisms of resistance that could lead to this phenomenon among offending strains. 

## 2. Methods

We conducted a retrospective cohort study at the Assaf Harofeh Medical Center (AHMC) in Israel from January 2010 to August 2014. Adult patients (≥18 years) with monomicrobial BSI (per established criteria [8]), resulting from a PA strain exhibiting non-susceptibility to either meropenem or imipenem (minimal inhibitory concentration (MIC) > 2 mg/L), and susceptibility to either ceftazidime (MIC < 16 mg/L), piperacillin (MIC < 32 mg/L), or piperacillin-tazobactam (MIC < 32/4 mg/L), were enrolled (following the Clinical and Laboratory Standards Institute (CLSI) criteria [9]). Patients with infectious syndromes necessitating prolonged treatment durations (e.g., endocarditis, osteomyelitis, and central nervous system infections) were excluded. Patients with repeated PA BSI were included only once in the analyses. We compared the outcomes of patients who received two or more doses of an appropriate (per in vitro report) beta-lactam agent (defined as “cases”), to those who received two or more doses of an appropriate non-beta-lactam regimen (defined as “controls”), from 48 h prior to the time blood cultures were drawn, to seven days following the culture date. Patients who received agents from both study arms (even a single dose) were excluded. Data were extracted from all available records, and post-hospitalization mortality data were captured from a national registry.

A *P. aeruginosa* strain was thawed and subcultured followed by manual extraction of genomic DNA. Minimal inhibitory concentrations (MICs) to imipenem and meropenem were confirmed with Etest^®^ (bioMérieux, Marcy-l’Étoile, France). Following preparation of genomic libraries with the Nextera XT kit according to manufacturer’s instructions, whole genome sequencing was performed using Illumina Nextseq paired ends 75 bp*2 aiming at coverage of at least 100× (Illumina, San Diego, CA, USA). Raw genomic output was analyzed using an in-house microbial pipeline at the Ben-Gurion University. Quality control was performed using FastQC and assembly and annotation were performed using SPAdes version 3.1.1 (Center for Algorithmic Biotechnology, St. Petersburg State University, Sankt-Peterburg, Russia) and Prokka version 1.11 (Torsten Seemann, Carlton, Australia), respectively. Relevant gene sequences were retrieved from the sequenced genome using BLAST. This included the extraction of each of the 7 loci used for determining the allelic profile and classifying the sequence type (ST) according to the international MLST scheme [10]. Detection of antimicrobial resistance genes was performed against the Comprehensive Antibiotic Resistance Database (CARD) [11] as well as ResFinder online tool [12]. Mutations and insertion/deletions of the *oprD* gene were analyzed in comparison to the PAO1 reference strains as previously described [13]. The whole genome sequence is available at Bioinformatics Core Facility [14].

## 3. Results

There were 67 unique patients with monomicrobial PA BSI that were non-susceptible to carbapenems but susceptible to the aforementioned cephalosporins and/or penicillins. This constituted 8.2% of all unique PA BSI cases during the 56-months study period. However, after excluding all patients who received one dose or more from both study arms, only 26 patients remained in the efficacy analyses cohort: 18 who were treated with an “older” beta-lactam: 9 with piperacillin-tazobactam, 7 with ceftazidime, and two patients received a combination of both agents (during the therapeutic time frame as displayed in the methods, but not at the same time). Eight patients were treated with non-beta-lactam regimens: three with a fluoroquinolone, two with colistin, one with an aminoglycoside and a fluoroquinolone, one with an aminoglycoside and colistin, and one with a fluoroquinolone and colistin. The cohort consisted overall, of elderly patients (mean age was 75 ± 11 years), with multiple comorbidities and healthcare exposures. Table 1 depicts patient characteristics and outcomes. Pneumonia was the most common clinical syndrome. 

There were no significant differences between the two study groups in terms of demographics, background conditions, healthcare exposures, or acute illness indices. The consolidative (main) regimen among survivors of the index hospitalization was administered for a median of 12 days (range 8–19) to cases and for 13 days (range 7–21) to controls (*p* = 0.6 between groups). In 8 cases (44%) and 5 controls (63%), additional PA were isolated (not necessarily from the blood) following the index isolation (OR = 0.5, *p* = 0.67). Among this group of eight case patients, none of the subsequent strains became resistant to the beta-lactam agent that the patient was receiving. 

All clinical outcomes were similar between groups, including mortality rates, length of stay among survivors, and additional hospitalizations following the index infection (Table 2). Seven Individual multivariable models were conducted for each of the outcome parameters as listed in Table 2: being a case or a control patient was not significantly associated with any of the analyzed outcomes.

The strain from patient number 3 (per Table 1) that was genomically analyzed was determined as ST137. The isolate was susceptible to piperacillin-tazobactam, had an MICs: above 16 mg/L for imipenem (resistant), 4 mg/L for meropenem (intermediate), less than 4 mg/L for ceftazidime (susceptible), less than 8 mg/L for ticarcillin (susceptible), and less than 4 mg/L for piperacillin (susceptible). The strain was also susceptible to gentamicin, amikacin, colistin, ciprofloxacin, and levofloxacin. Interrogation of the genome did not reveal any potent carbapenemases. However, several resistance determinants were detected; specifically, several families of efflux pumps (i.e., mexA, mexC, mexE, mexG, mexH, mex J, mexL, mexM, mexP, mexS, mexV, mexW and mexX, mdtC/mexN, oprM, and oprN)) and beta-lactamases, including OXA-50 and chromosomally mediated Pseudomonas-derived cephalosporinases (PDC). The oprD gene appeared to be intact with 100% sequence homology to the reference strain. 

## 4. Discussion

Treatment of PA infections is limited, specifically due to its extensive ability to evade antimicrobial agents via multiple pathways. The “older” beta-lactams are bactericidal, safe, and (usually) inexpensive, with established efficacy against PA infections. Therefore, they are considered the agents of choice for susceptible isolates [3]. Group 2 carbapenems are broad-spectrum beta-lactams and are frequently used in nosocomial settings for severe invasive PA infections. Many of the common mechanisms of resistance to carbapenems in PA (specifically, the carbapenemases which are prevalent and more potent compared to other mechanisms), pose a ‘class effect’ against the whole spectrum of beta-lactams [4]. However, there are other mechanisms of resistance which are specific to certain carbapenems, but not to the whole class of beta-lactams (e.g., certain efflux pumps and mutations resulting in decreased expression of outer membrane proteins) [4]. These mechanisms are usually less potent [4], and may even target only certain carbapenems [4]. Since some of the inductive mechanisms of resistance to carbapenems and beta-lactams are chromosomally encoded [4], it is uncertain whether it is safe to treat severe invasive PA infections, resistant to a carbapenem but susceptible to an “older” anti-Pseudomonal penicillin (e.g., piperacillin) or cephalosporin (e.g., ceftazidime), with a non-carbapenem beta-lactam alternative. These phenotypes are becoming more and more prevalent, constituting ~10% of PA-BSI strains in many institutions worldwide. Additionally, it is unknown whether resistance to all beta-lactams could emerge while the patient is being treated with a ‘narrow-spectrum’ beta-lactam, even for shorter treatment courses (i.e., up to 14 days). On the contrary, non-beta-lactam alternatives could be more toxic and less effective when compared to beta-lactams. 

In this small retrospective analysis, 26 patients were enrolled over a 4.5-year period at a single medical center in central Israel. The resistance phenotype was more common than the actual number of patients enrolled in the study. However, many patients received at least a single dose from both study arms and therefore, were excluded from the efficacy analysis. In this analysis, there were no differences in a variety of measured outcomes, including various morbidity and mortality indices. The length of stay following the isolation among survivors was also not affected by the administered therapeutic regimen. In addition, there were no additional isolations of PA strains that became resistant to all beta-lactam agents, among the patients who were treated with non-carbapenem beta-lactams. Therefore, we suggest that if a patient isolate is non-susceptible to a carbapenem, but is susceptible in vitro to another β-lactam (for treatment courses that last up to 14 days), it is reasonable to use “older” penicillins or cephalosporins. We acknowledge that larger prospective interventional studies are needed in order to validate this recommendation. 

Phenotypic features of strains suggest that this was not a clonal cluster or outbreak of a certain strain and that multiple mechanisms probably played a role in the resulting general phenotype (i.e., non-susceptibility to one or more carbapenems and susceptibility to one or more non-carbapenem “older” beta-lactam penicillin or cephalosporin). In the genomically analyzed strain, a combination of beta-lactamases, coupled with efflux pumps, was identified and probably underlines the mechanism to the high MIC to imipenem, as previously described elsewhere [15]. OXA-50, a weak beta-lactamase that may increase MIC to imipenem, may also have contributed to this resistance phenotype [16]. There was no in silico evidence for absent or mutated porin protein (oprD), which has been previously described as being the most common mechanism associated with carbapenem non-susceptibility [17]. However, we did not perform gene expression studies and therefore, a loss of porin cannot be ruled out. More such isolates from other centers should be studied, sequenced, and analyzed, in order to elucidate the existing mechanisms of resistance that are responsible for this increasingly prevalent, yet clinically challenged phenomenon.

## Figures and Tables

**Table 1:** Clinical and microbiological characteristics of patients with *Pseudomonas aeruginosa* bloodstream infection that are non-susceptible to carbapenems but susceptible to cephalosporins and/or to penicillins, Assaf Harofeh Medical Center (January 2010 to August 2014).

No	Age	IMI MIC	MERO MIC	CTZ MIC	PIP MIC	P/TZ “Status”	COL MIC	AMK MIC	GEN MIC	CIP MIC	Syndrome	Hours to Appropriate Rx	Main Rx	Death within 14 Days	Death within 30 Days	Death within 90 Days	LOS
1	82	8	8	4	8	S	2	1	2	0.125	IAI	0	P/TZ	No	No	No	11
2	69	8	4	4	2	S	2	1	0.5	0.125	Pneumonia	0	CTZ	No	Yes	Yes	
3	54	32	4	0.5	2	S	2	1	0.5	0.125	IAI	53	CTZ	Yes	Yes	Yes	
4	59	32	32	4	16	S	2	16	32	8	IAI	64	CTZ&PIP	No	No	No	10
5	93	32	32	16	256	S	2	128	32	8	UTI		P/TZ	No	Yes	Yes	
6	67	8	4	2	16	S	1	1	2	0.125	Pneumonia	36.5	CTZ	Yes	Yes	Yes	
7	72	32	32	4	16	S	1	1	0.5	1	Pneumonia	0	CTZ	Yes	Yes	Yes	
8	79	32	32	8	256	R	2	128	32	8	Pneumonia		P/TZ	Yes	Yes	Yes	
9	78	16	4		64	S	2	8	4	2	Primary BSI	61	P/TZ	No	No	No	27
10	64	32	4	0.5	2	S	2	1	4	0.125	Pneumonia	19	P/TZ	No	No	No	39
11	74	32	32	8	16	S	2	4	4		Primary BSI	0	P/TZ&CTZ	No	No	No	32
12	71	32	4	0.5	2	S	0.25	1	0.5	0.125	SSTI	0	P/TZ	Yes	Yes	Yes	6
13	84	32	32	4	16	S	0.25	16	32	8	UTI	0	CTZ	No	No	No	8
14	79	8	8	4	256	R	0.25	16	32	8	Pneumonia	62.5	CTZ	No	No	Yes	34
15	55	16	8	16		S					Pneumonia	0	P/TZ	No	Yes	Yes	
16	88	4	32	8	64	S	0.25	1	8	8	Pneumonia	83	P/TZ	Yes	Yes	Yes	
17	90		32	4	16	S	0.25	1	0.5	8	Pneumonia	79	CTZ	No	Yes	Yes	
18	96		32	4	64	S	0.25	1	8	8	UTI	6	P/TZ	No	No	No	10
19	62	8	32	16	64	S	2	1	0.5	1	Primary BSI	0	GEN&CIP	No	No	No	14
20	76	32	32			S					Pneumonia	72	AMK&COL	No	Yes	Yes	
21	76	32	4	8	2	S	1	1	0.5	0.125	Pneumonia	4	LEV	No	Yes	Yes	
22	64	8	1	2	8	S	0.25	1	0.5	0.125	UTI	60	CIP	No	No	No	5
23	81	32	8	4	256	R	0.25	16	32	8	Pneumonia	0	LEV&COL	Yes	Yes	Yes	
24	81	32	2	2	2	S	0.25	1	2	0.125	IAI	91	CIP	Yes	Yes	Yes	5
25	86	32	8	4	256	R	2	16	32	8	UTI	50	COL	No	No	No	23
26	78	32	32	128	256	S	0.25	128	32	8	Primary BSI	1.5	COL	No	No	No	

Note: The first 18 patients are the “cases” treated with a non-carbapenem beta-lactam and the last 8 patients are the “controls” treated with a non-beta-lactam based regimen. The susceptibility “status” for piperacillin/tazobactam is based of the Clinical and Laboratory Standards Institute (CLSI) criteria [9]. No. = patient number. MIC = minimal inhibitory concentration (mg/L). Rx = therapy. S = susceptible. R = non-susceptible. IAI = intra-abdominal infection. UTI = urinary tract infection. BSI = bloodstream infection. SSTI = skin and soft-tissue infection. LOS = the length of stay, in days, after excluding the patients who died. IMI = imipenem. MERO = meropenem. CTZ = ceftazidime. PIP = piperacillin. P/TZ = piperacillin/tazobactam. COL = colistin. AMK = amikacin. GEN = gentamicin. CIP = ciprofloxacin. LEV = levofloxacin.

**Table 2:** Efficacy analysis of non-carbapenem beta-lactams versus non-beta-lactams for treating carbapenem non-susceptible *Pseudomonas aeruginosa* bloodstream infections.

Outcome Parameter	Beta-Lactam Rx (*n* = 18)	Non-*β*-Lactam Rx (*n* = 8)	OR	CI-95%	*p* Value
In hospital mortality	11 (61)	4 (50)	1.6	0.3–8.4	0.70
14 days mortality	6 (33)	2 (25)	1.5	0.2–9.8	>0.99
30 days mortality	10 (57)	4 (50)	1.3	0.2–6.6	>0.99
90 days mortality	11 (61)	4 (50)	1.6	0.3–8.4	0.68
Functional deterioration	4 (57)	0			0.2
Additional hospitalizations in 3 months following the index infection	11 (92)	5 (83)	2.2	0.1–43	>0.99
LOS median (IQR)	11 (6–39)	11 (5–23)			0.30

Data are reported as number (valid percent. i.e., excluding missing information from the denominator) unless otherwise specified. Note: Rx = therapy. OR = odds ratio. CI = confidence interval. LOS = length of hospital stay from infection to discharge after excluding the patients who died in hospital. IQR = interquartile range.
